# Safety and Efficacy of Transvaginal Natural Orifice Transluminal Endoscopic (vNOTES) Right Colectomy: A Systematic Review

**DOI:** 10.3390/cancers17162699

**Published:** 2025-08-19

**Authors:** Georgia Dimopoulou, Konstantinos Perivoliotis, Evangelos Lolis, Dimitrios Symeonidis, Konstantinos Tepetes, Ioannis Baloyiannis

**Affiliations:** 1 Department of Surgery, “Achillopouleion” General Hospital, 382 22 Volos, Greece; geodim14@hotmail.com; 2 Department of Colorectal Surgery, The Royal Marsden NHS Foundation Trust, London SW3 6JJ, UK; 3 Department of Surgery, University Hospital of Ioannina, 455 00 Ioannina, Greece; vlolis@uoi.gr; 4 Department of Surgery, University Hospital of Larissa, 413 34 Larissa, Greece; simeonid@hotmail.com (D.S.); tepetesk@gmail.com (K.T.); balioan@hotmail.com (I.B.)

**Keywords:** transvaginal, right, colectomy, notes, vaginal

## Abstract

**Background/Objectives**: We aim to provide pooled data on the safety and efficacy of Transvaginal Natural Orifice Transluminal Endoscopic (vNOTES) right colectomy. **Methods**: This systematic review was conducted according to the Cochrane Handbook and the Preferred Reporting Items for Systematic Reviews and Meta-Analyses (PRISMA) guidelines. The primary endpoint was the overall complication rate. Quality assessment was based on the NHLBI quality assessment tools. **Results**: Overall, six studies were included in this review. Overall morbidity rate was 21.9% (95% CI: 10.7–33.2%, *p* < 0.001), while intraoperative adverse events were noted in 19.9% (*p* < 0.001) of cases. Mean operation duration was 176.42 (*p* < 0.001) minutes. Overall hospital stay was 8.68 days (*p* = 0.002). **Conclusions**: Our analyses confirm the safety and efficacy of the approach. Given several study limitations, further large-scale and high-quality trials are required.

## Simple Summary

Currently, there is absence of pooled evidence regarding the overall morbidity, the postoperative recovery profile, and the oncological efficiency of vNOTES right colectomy. Our study highlighted an acceptable rate of overall and intraoperative complications of this technique. The efficacy of the approach has been highlighted on several efficiency markers, including the operation duration and the length of hospital stay.

## 1. Introduction

### 1.1. Rationale

The advent of minimally invasive techniques marked a paradigm shift in surgical practice that resulted in improved cosmesis, reduced postoperative pain, and enhanced aspects of postoperative recovery [1,2,3]. In addition to laparoscopic and robotic surgeries, single-incision approaches were also described as a means of further minimizing total transabdominal entrance points to one [4,5].

Natural Orifice Translumenal Endoscopic Surgery (NOTES) is another alternative that is considered by many as the natural sequalae of single-incision techniques [6]. However, the combination of technical challenges and a steep learning curve have prohibited a wider adoption of NOTES [7,8].

Similarly, in the domain of colorectal surgery, natural orifice approaches have been utilized for specific procedural steps, such as dissection and specimen retrieval, or the completion of the operation in total [6,8,9]. Typical examples are the trans-anal total mesorectal excision (TaTME) and the transvaginal extraction of colectomy specimens (natural orifice specimen extraction (NOSE)) [6,7,10].

For female patients with right colonic pathology, transvaginal NOSE was shown in multiple reports to be a valid option for opting out of an abdominal incision, thus augmenting patient satisfaction and attenuating wound-related morbidity [11,12]. On the other hand, due to anatomical restrictions, alteration of the operative field of view, and single-port related loss of triangulation and instrument clashing, Transvaginal NOTES (vNOTES) right colectomy represents a major leap in terms of operative difficulty [9,13]. This is clearly depicted by the fact that the existing literature on vNOTES right colectomy largely comprises a relatively small number of case series and early feasibility studies, with varying methodologies and outcome measures [9,14,15]. Additionally, to the best of our knowledge, there is currently no pooled evidence regarding the overall morbidity, the postoperative recovery profile, or the oncological efficiency of this technique.

### 1.2. Aim

The present study aims to critically synthesize the current data regarding the safety and efficacy of vNOTES right colectomy, thereby elucidating the potential role of this approach in the surgical management of right-sided colonic pathology.

## 2. Materials and Methods

### 2.1. Study Protocol

This systematic review was conducted in accordance with the Cochrane Handbook and the Preferred Reporting Items for Systematic Reviews and Meta-Analyses (PRISMA) guidelines [16,17].

The study protocol has been registered (https://doi.org/10.17605/OSF.IO/JEZX8).

### 2.2. Search Strategy

A comprehensive search strategy was applied across major databases to identify eligible studies. Screening was performed in PubMed, Scopus, and Cochrane databases from inception until March 2025. The following keywords were used, combined with Boolean logic nexuses:

“vaginal”, “transvaginal”, “natural orifice”, “notes”, “right colectomy”, “right colon”

Reference screening was also performed in the eligible articles.

### 2.3. Endpoints

The primary endpoint of our study was the overall complication rate. Secondary outcomes included specific intraoperative (i.e., hemorrhage, blood loss, bladder injury, conversion) and postoperative adverse events (hematoma, ileus, vaginal infection, bacteremia, anastomotic bleeding). Further analyses were performed in efficacy (operation duration, length of hospital stay, mobilization, and time to first flatus) and oncological (recurrence rates and lymph node yield) points of interest.

### 2.4. Eligibility and Exclusion Criteria

All human studies reporting on adult patients submitted to vNOTES right colectomy and providing data on outcomes of interest were considered as eligible.

The following exclusion criteria were considered: (1) non-human studies, (2) studies not reporting data on outcomes of interest, (3) pediatric population, (4) articles in the form of editorials, letters, or conference abstracts, and (5) studies in which transvaginal access was used for specimen extraction only (NOSE).

### 2.5. Quality Assessment

The methodology used to assess quality was structured around standardized checklists tailored to different study designs: case reports, prospective cohorts, and case series. For each type, a set of specific criteria was defined based on the National Heart, Lung, and Blood Institute (NHLBI) quality assessment tools. Each study was systematically evaluated against these criteria. This approach ensures a transparent and consistent evaluation of study quality, highlighting strengths and weaknesses in reporting and design.

### 2.6. Study Selection and Data Collection

After the completion of database screening, duplicate entries were removed. Following title and abstract screening, the remaining records were submitted to a full text review to evaluate consistency with the predefined eligibility criteria. Data extraction and quality assessment were performed independently and blindly by two reviewers (G.D. and I.B.). In the case of a discrepancy that was not resolved through mutual discussion, the opinion of a third reviewer (K.P.) was considered.

From the eligible studies, data regarding study characteristics (first author, date of publication, type of study, country, number of involved centers, study period, number of patients, body mass index (BMI), age, and follow up period), patient and tumor characteristics (previous operations, American Society of Anesthesiologists (ASA) score, Tumor Node Metastasis (TNM) status, and tumor location), and technical operative details (preoperative bowel preparation, patient position, pneumoperitoneum, access, number of trocars, anastomosis technique, approach, number of surgeons, and access closure) were extracted. Additionally, data regarding the prespecified outcomes were recorded.

### 2.7. Statistical Analysis

All statistical analyses were performed in IBM SPSS version 29 and Open Meta Analyst. Continuous and categorical data were provided as mean (standard deviation (SD)) and N, respectively. In the case that these were not provided, they were estimated from the respective data (median, range, interquartile range (IQR)), using the algorithm proposed by Hozo et al. [18]. Moreover, combined group means and SDs were calculated [16].

Pooled continuous outcomes were reported as mean, with the corresponding 95% confidence interval (95% CI). The effect size of binary outcomes was the raw proportion (RP), with the 95% CI. For the identification of publication bias, the respective funnel plot of the primary endpoint was provided.

Statistical analysis was based on the DerSimonian–Laird method. Heterogeneity was estimated through the calculation of I^2^, while Cochran Q test results confirmed the significance. The random-effect (RE) and fixed-effect (FE) models were applied based on the estimated significance. Statistical significance was considered at the level of *p* < 0.05.

## 3. Results

### 3.1. Search Results

**Figure 1:**
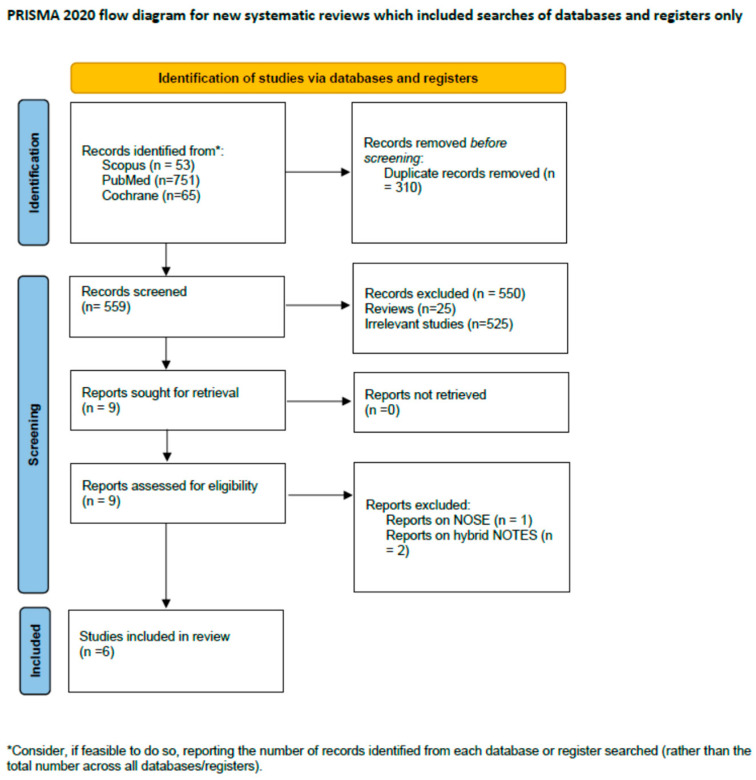
PRISMA flow diagram.

An initial literature search (Figure 1) identified 869 records. After the removal of 310 duplicates, 559 titles and abstracts were screened. During the first screening step, 550 records (25 reviews and 525 irrelevant studies) were excluded. Subsequently, nine manuscripts were retrieved and underwent a full-text assessment. One study was excluded due to reporting on NOSE procedure, and two [13,14] for providing data on hybrid NOTES. Two studies were identified to be conducted in the same research center with similar methodology, as well as inclusion and exclusion criteria [15,19]. However, due to not totally overlapping study periods and the differences in patient characteristics, both were included. Overall, six studies [9,15,19,20,21,22] were included in this review.

### 3.2. Study Characteristics

Overall, 49 patients that underwent vNOTES right colectomy were included in this review (Table 1). In total, five studies [15,19,20,21,22] were performed in a single institution, and one [9] in multiple centers. Most eligible studies reported on performed vNOTES right colectomies in the form of individual case reports or case series [20,21,22]. Study periods ranged from 2006 to 2024. BMI and age allocation of included patients is also provided in Table 1. Mean postoperative follow-up ranged from 1 to 60 months.

Data regarding further patient and tumor characteristics are also provided in Appendix A Table A1 and Table A2. In terms of technical characteristics, all procedures were performed in the lithotomy position, with a pneumoperitoneum pressure ranging from 12 to 14 mmHg. All cases were performed laparoscopically, and multiple transvaginal single-access ports were used. Significant heterogeneity was noted in terms of the number of transvaginal trocars. The use of abdominal accessory trocars was reported in two studies. Dissection was performed in a medial to lateral or an inferior to superior approach. Both intracorporeal and extracorporeal anastomoses were described. Information regarding the number of operating surgeons and experience was scarce. In all reported cases, the access closure was performed with direct suturing.

### 3.3. Quality Assessment

A structured quality assessment (Appendix A Table A3, Table A4 and Table A5) was conducted using standardized checklists tailored to the respective study design—case report or prospective cohort. Among the case reports, one study [22] achieved a perfect score of 100%, while the rest demonstrated acceptable-quality grades (87.5%). For cohort studies, quality scores ranged from 78.6% to 85.7%.

### 3.4. Primary Outcomes

**Figure 2:**
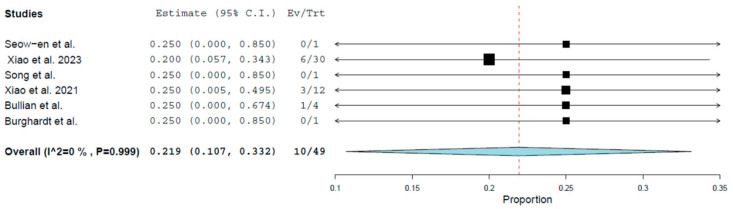
Overall complication forest plot.

**Figure A1:**
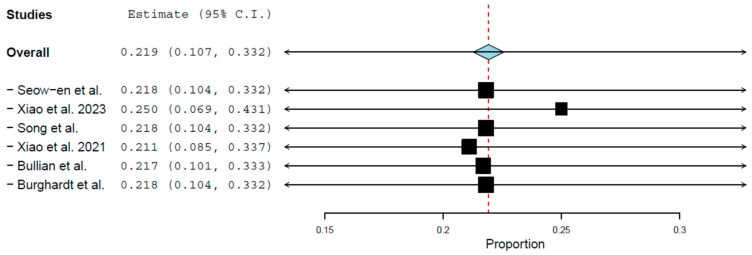
Leave one out analysis of the primary outcome.

The pooled complication rate of vNOTES right colectomy (Table 2, Figure 2) was 21.9% (95% CI: 10.7–33.2.0%, *p* < 0.001). No significant heterogeneity was noted (I^2^ = 0%). The effect of each study was evaluated through a leave-one-out analysis (Appendix A Figure A1). The pooled estimate ranged from 21.1% (Xiao et al. 2021 [15]) to 25% (Xiao et al. 2023 [19]). Statistical significance of the pooled results and the heterogeneity levels were retained in all cases, respectively.

### 3.5. Secondary Outcomes

**Figure A2:**
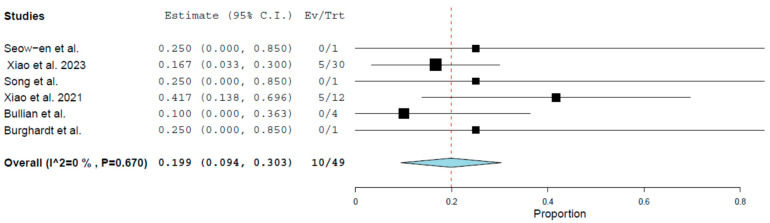
Intraoperative Complications Forest Plot.

**Figure A3:**
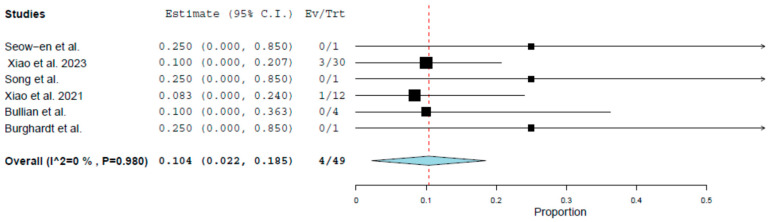
Intraoperative Bladder Injury Forest Plot.

**Figure A4:**
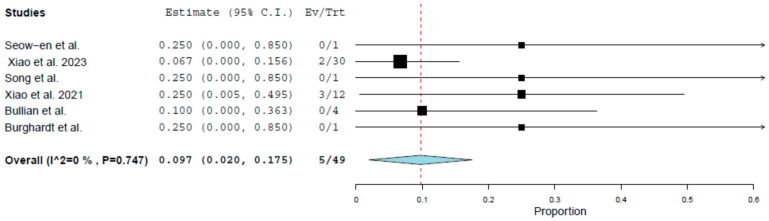
Intraoperative Hemorrhage Forest Plot.

**Figure A5:**
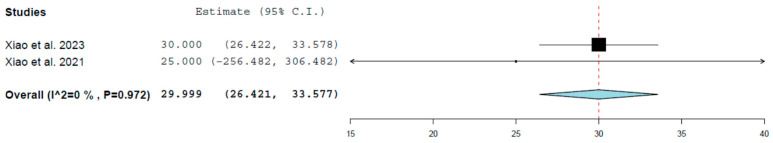
Intraoperative Blood Loss (mL) Forest Plot.

**Figure A6:**
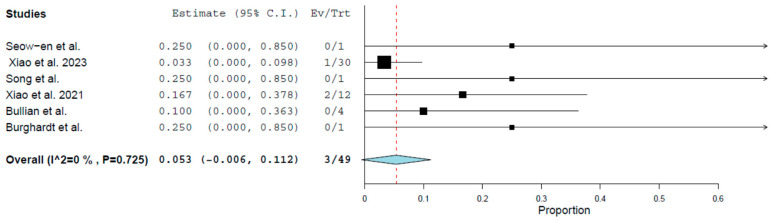
Conversion Forest Plot.

Similarly, the intraoperative complication rate (Appendix A Figure A2) was 19.9% (95% CI: 0.9–30.3%, *p* < 0.001). Of these, the most common was intraoperative bladder injury (Appendix A Figure A3), with an overall risk of 10.4% (95% CI: 2.2–18.5%, *p* = 0.013). Although significant hemorrhage (Appendix A Figure A4) was reported in 9.7% (95% CI: 2–17.5%, *p* = 0.014) of cases, the mean intraoperative blood loss (Appendix A Figure A5) was 29.9 mL (95% CI: 26.42–33.57 mL, *p* < 0.001). Conversion (Appendix A Figure A6) due to technical difficulties was required in 5.3% (95% CI: −0.6–11.2%, *p* = 0.076) of the procedures; however, this was not significant.

**Figure A7:**
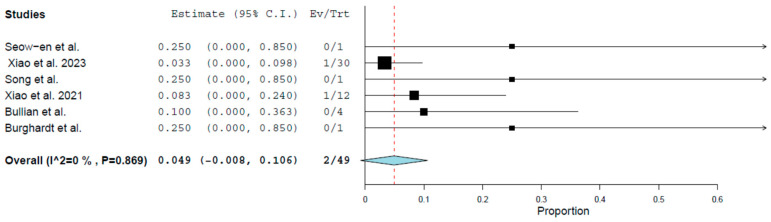
Postoperative Hematoma Forest Plot.

**Figure A8:**
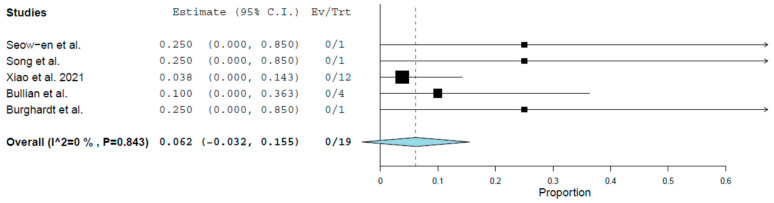
Postoperative Ileus Forest Plot.

**Figure A9:**
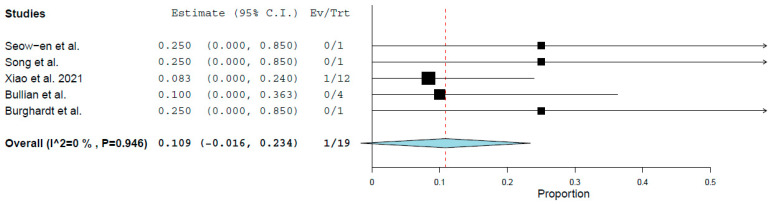
Postoperative Vaginal Infection Forest Plot.

**Figure A10:**
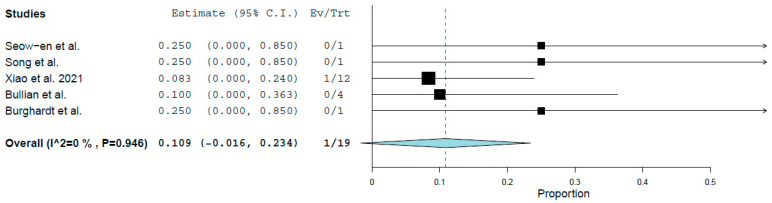
Postoperative Bacteremia Forest Plot.

**Figure A11:**
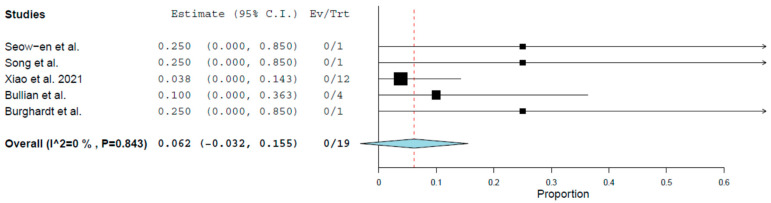
Postoperative Anastomotic Bleeding Forest Plot.

**Figure A12:**
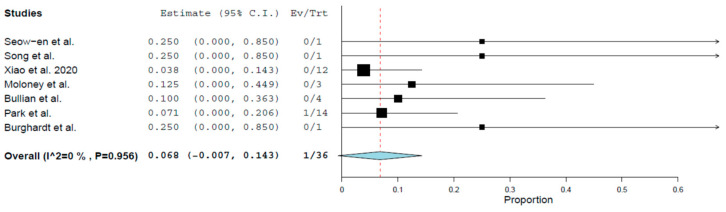
Recurrence Forest Plot.

**Figure A13:**
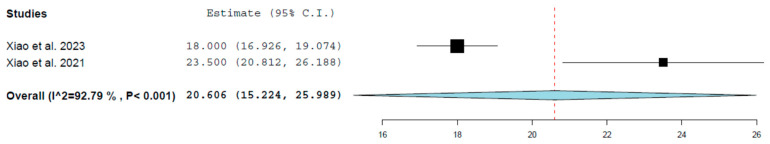
Lymph Node Yield Forest Plot.

The pooled rates of specific postoperative complications, including hematoma (Appendix A Figure A7, 4.9% 95%CI: −0.8–10.6%, *p* = 0.093), ileus (Appendix A Figure A8, 6.2% 95%CI: −3.2–15.5%, *p* = 0.196), vaginal infection (Appendix A Figure A9, 10.9% 95%CI: −1.6–23.4%, *p* = 0.088), bacteremia (Appendix A Figure A10, 10.9% 95%CI: −1.6–23.4%, *p* = 0.088) and anastomotic bleeding (Appendix A Figure A11, 6.2% 95%CI: −3.2–15.5%, *p* = 0.196) did not reach statistical significance. Furthermore, an insignificant 6.2% (Appendix A Figure A12, 95%CI: −3.2–15.5%, *p* = 0.196) rate of tumor recurrence was estimated. Mean lymph node yield (Appendix A Figure A13) was 20.6 (95% CI: 15.2–25.9, *p* < 0.001).

**Figure A14:**
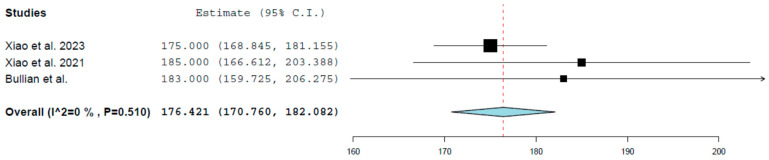
Operation Duration (minutes) Forest Plot.

**Figure A15:**
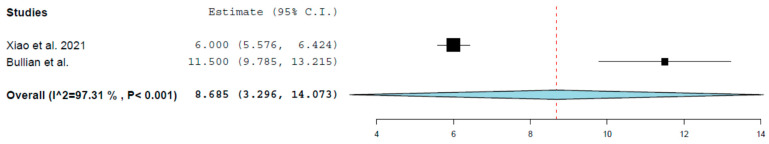
Length of Hospital Stay (days) Forest Plot.

Mean operation duration (Appendix A Figure A14) was 176.42 min (95% CI: 170.76–182.08, *p* < 0.001). The reported overall mean hospital stay (Appendix A Figure A15) was 8.68 days (95% CI: 3.29–14.07, *p* < 0.001). Data regarding patient mobilization and the time to first flatus were scarce and, thus, no further analysis was performed. More specifically, patients mobilized at 18 to 24 h; while regarding the latter, first flatus was achieved at 24 to 40 h postoperatively.

### 3.6. Publication Bias

**Figure 3:**
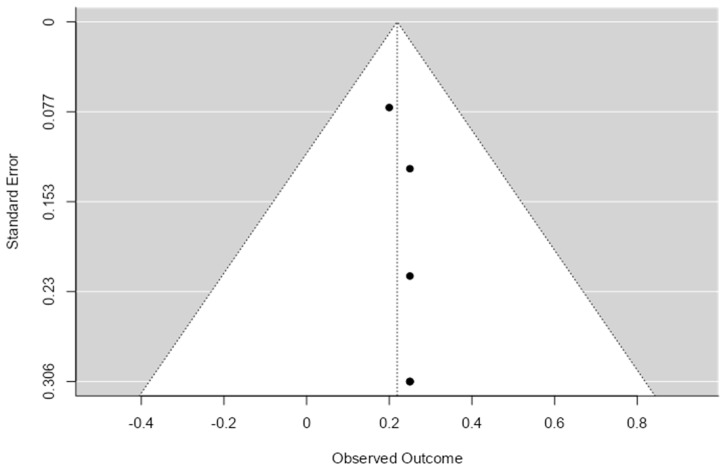
Overall complication funnel plot.

To evaluate potential publication bias, we generated the primary outcome funnel plot (Figure 3). Visual inspection of the plot revealed a symmetrical distribution of the eligible studies, thus minimizing the risk of publication bias.

## 4. Discussion

The description of NOTES further pushed the boundaries of minimally invasive colorectal surgery [13,14,15]. Due to its elasticity, expedited healing, and optimal cosmesis, the transvaginal route was initially used for specimen retrieval, thus abolishing the need for transabdominal incisions [11,12]. Further evolvement of this conception was vNOTES, in which mobilization, vessel ligation, and colonic transection are performed through the vagina [11,12].

To introduce the camera and the working instruments during vNOTES, a transvaginal single-port device is utilized [15]. However, due to the proximity and the interference between ports, dissection is impeded [15]. Another important technical difficulty is the differentiation of the surgical visualization compared to in other minimally invasive modalities [15]. In laparoscopic, single-incision, and robotic approaches, a top-down view of the surgical field is achieved; however, in vNOTES, the camera is inserted from a lower pivotal point, thus resulting in a horizontal view. Subsequently, for a complete mesocolic excision to be performed, alteration of the dissection strategy may be required [21,22].

It becomes apparent that the safety of vNOTES right colectomy should be carefully examined prior to its widespread adoption. We estimated a pooled overall complication rate of vNOTES right colectomy of 21.9%, with bladder injury being the most common. In an 8257-patient meta-analysis, Solaini et al. [23] compared the two most prominent minimally invasive techniques for right colonic surgery and estimated the mean morbidity rates of the laparoscopic and robotic approaches to be 23.4% and 21.4%, respectively.

To tackle the previously mentioned technical difficulties, and to facilitate proper exposure of the embryological planes, assistant ports or hybrid vNOTES approaches may be utilized [15]. However, in some cases, this may not be achieved, and a standard laparoscopic or open conversion may be required. Our pooled analysis estimated an insignificant 5.3% overall conversion rate of vNOTES. Comparably, according to previous publications, the conversion rate of single-incision right colectomy may reach the level of 7.4% [23].

Early publications raised significant concerns regarding the morbidity related to intra-abdominal bacterial seeding from the opening of a natural orifice during NOTES [13]. However, in a recent systematic review by Li et al. [24], these risks were shown to be minimal. Similarly, in a feasibility cohort by Xiao et al. [15], one patient out of twelve developed vaginal discharge, and one developed bacteremia. We calculated the pooled vaginal complications of vNOTES right colectomy to be non-significant, at 10.9%.

Even though operative time can be affected by multiple parameters, including case complexity, technical competency, and theater personnel coordination, it still is one of the most important efficacy metrics, with a direct impact on clinical and logistic outcomes [25]. In a network meta-analysis by Rausa et al. [26], the mean operative times between the different minimally invasive approaches were not significantly different. Interestingly, Liu et al. [27] reported that single-incision techniques required a mean 129 to 217 min, 23.49 min shorter compared to standard laparoscopic right colectomies. We estimated that the mean operation duration of vNOTES right colectomy was 176.42 min. Although we did not perform pairwise comparisons with other modalities, the estimated effect size is within the reported range from other publications.

The adoption of the ERAS protocols promoted the enhancement of postoperative recovery, thus increasing patient satisfaction and minimizing of hospitalization costs [28]. Minimization of incisions is among the various technical interventions described in these protocols [28]. In other minimally invasive techniques for right colectomy, the first flatus landmark is achieved at 2.3–3.3 days postoperatively, and patients are discharged after 5.8–6.1 days [29]. Regarding vNOTES, due to lack of data, pooled evidence could not be provided.

In terms of oncological efficacy, we estimated that the average lymph node yield of vNOTES colectomy was 20.06. In addition to this, the estimated recurrence rate during the analyzed follow-up was 6.2%; however, this was not statistically significant. These results are consistent with the current literature regarding the oncological endpoints of minimally invasive right colectomies. More specifically, in a systematic review by Apostolou et al. [30], the number of harvested lymph nodes in single-incision right colectomy was 19.2. Similarly, Stipa et al. [11] estimated that, following laparoscopic NOSE colorectal resections, the mean lymph node yield was 12, with no evidence of disease recurrence. The oncological efficacy of transvaginal approaches was further confirmed in the meta-analysis by Chang et al. [31]. In this study, transvaginal and transabdominal specimen extraction displayed similar 2- and 3-year disease free survival, with no reports of pelvic or vaginal seeding.

The main advantage of the vNOTES approach is minimization of the need for a transabdominal incision. Through a posterior colpectomy and a single-port device, functional access to the abdominal cavity is installed [15]. In pure vNOTES, the entire operation is performed through the single-port device, whereas, in hybrid techniques, only certain procedural steps are completed transvaginally [13,14]. The importance of these lies to the extent of the required abdominal incision. In the former, no transabdominal access is utilized, whereas, in the latter, standard laparoscopic ports may be inserted. The reduction in the cumulative length of a surgical wound is important due to multiple reasons. First, due to the association between surgical injury and the inflammatory cascade, a decrease in incision length directly improves multiple clinical parameters [32]. Second, reducing the length of the incision promotes cosmesis, increases patient satisfaction, and enhances patient well-being [33]. Finally, transabdominal incisions are associated with significant complications, such as the development of infection, seroma, wound dehiscence, and incisional hernias that may eventually require reoperation [34]. Therefore, by utilizing a natural orifice access, the risk for such adverse events is avoided.

Another sequalae of abdominal wounds is postoperative pain [33]. Through laparoscopic and robotic resections, incision wounds were minimized to port placement and specimen extraction, thus decreasing postoperative pain compared to open resections [26]. Less pain translates to earlier mobilization, better functional recovery, reduced risk for respiratory compromise, and overall patient acceptance [33]. The use of the vaginal natural orifice to either extract the specimen or complete the operation would theoretically abate postoperative pain scores. In a prospective randomized controlled trial by Leung et al. [35], hybrid NOTES colectomy for left-side tumors had significantly lowered maximum pain scores during the first week, compared to conventional laparoscopic colectomy. Moreover, in a meta-analysis by He et al. [36], NOSE application in colorectal resections led to reductions in postoperative pain and prescribed analgesics. In our review, pooled postoperative pain estimates were not provided, due to the scarcity of data. Visual analog scale scores during the first postoperative days were reported in some cases and never exceeded the 4-point threshold.

Furthermore, concerns regarding sexual function and long-term pelvic floor outcomes remain insufficiently addressed. Most eligible records were case series and prospective or retrospective cohorts, with no distinct methodology for the assessment of these respective endpoints with objective or patient-reported validated tools. Therefore, until more evidence is available, careful patient selection and informed consent remain critical for incorporating this novel technique into clinical practice.

Optimal patient selection is of paramount importance when attempting to introduce vNOTES for the management of colorectal tumors. In addition to general and colorectal-specific medical history, the attending physician should also evaluate, in detail, the patient’s gynecological history [14]. According to a 2021 consensus [37] on the safe implementation of vNOTES, several exclusion criteria were proposed, including history of rectovaginal endometriosis and severe pelvic inflammatory disease. Additionally, history of pelvic radiotherapy was also suggested as an important factor for not implementing vNOTES [37]. On the other hand, the consensus statement did not identify nulliparity, previous caesarian section, or high BMI as contraindications for vNOTES [38]. In a cohort study by Park et al. [14], in which a hybrid vNOTES right colectomy was performed, bulky tumors > 5 cm, severe pelvic adhesions, history of endometriosis, and patients at child-bearing age were excluded. The rationale was that vaginal narrowing might prohibit the establishment of peritoneal access and specimen removal, while extensive pelvic adhesions increase the risk for adjacent organ injury [14]. Similar criteria were also used by Xiao et al. [15], who suggested a 6 cm tumor size cut-off.

Finally, another important consideration is the learning curve and technical prerequisites of vNOTES right colectomy. Surgeons require advanced laparoscopic skills and experience with Natural Orifice Transluminal Endoscopic Surgery (NOTES) techniques, which may limit the procedure’s widespread adoption [38,39]. As shown in various settings, reaching proficiency in NOTES requires the performance of a significant number of cases [40,41]. In terms of colorectal surgery, data is notably inconsistent [42]. For instance, in a review by Lau et al. [42], the number of procedures required to reach stabilization of the TaTME learning curve ranged from 5 to 140 cases. Additionally, evidence for the vNOTES right colectomy learning curve is currently scarce. More specifically, in our study, the number of experienced operating surgeons was not systematically reported.

Strengths

This systematic review is the first to provide overall estimates for vNOTES right colectomy performance. Our analyses assessed multiple clinical parameters that represent aspects of safety, perioperative efficiency, and oncological efficacy. The utilization of a standardized methodology allowed us to combine the data from the current literature reports and provide an accurate overall estimate on these endpoints. These indicators could act as guidance for clinicians evaluating the potential role of vNOTES techniques in right colon cancer. Finally, our review highlighted the lack of evidence in several clinical parameters, thus promoting further research in this field.

Limitations

Prior to the appraisal of our results, several study limitations should be considered. First, most trials reporting on vNOTES right colectomy were either individual case reports or cohort studies, with minimal sample sizes. Subsequently, the lack of blinding in assessing outcomes alongside specific methodology deficits significantly impacts the overall quality of evidence. Additionally, it was noted that there was no standardization of the approach, with multiple variances in the applied technique, thus reducing the reproducibility of our results. Moreover, the discrepancies in terms of patient and underlying pathology characteristics further reduce the ability to extrapolate our findings to a wider surgical population. In addition, despite a thorough assessment of the included studies, two of them were conducted in the same research center during partially overlapping periods, thus posing the risk of duplicate data. Furthermore, the lack of systematic long-term follow-up could possibly impact the results of oncological endpoints. Similarly, the absence of comparative data does not allow us to safely reach conclusions regarding the performance of vNOTES over other traditional minimally invasive techniques. Finally, surgical outcomes are significantly affected by the experience of the surgeons, and, thus, insufficient relevant data may lead to unsafe conclusions.

## 5. Conclusions

To the best of our knowledge, this study is the first attempt to provide pooled evidence regarding the safety and efficacy of vNOTES right colectomy. Our study highlighted an acceptable rate of overall and intraoperative complications. Additionally, the results of our analyses on several efficiency markers, including the operation duration, the length of hospital stay, and postoperative recovery endpoints, validated the efficacy of the approach. However, due to several study limitations, further high-quality trials are required to standardize the surgical technique and provide comparative data with other minimally invasive approaches.

## Figures and Tables

**Table 1:** Main characteristics of the included studies.

First Author	Publication Date	Type of Study	Country	Single-/Multi-Center	Study Period	Number of Patients	BMI	Age	Follow Up (Months)
Seow-en et al.	2024	case report	Singapore	single	2024	1	32 (0)	59 (0)	60 (0)
Xiao et al.	2023	retrospective	China	single	2019–2022	30	22 (3.1)	n/a	n/a
Song et al.	2021	case report	China	single	2021	1	18.4 (0)	65 (0)	1 (0)
Xiao et al.	2021	prospective	China	single	2018–2020	12	n/a	70 (7.5)	30 (0)
Bullian et al.	2014	prospective	Germany	multi	2008–2013	4	26 (2.75)	63.5 (5.5)	n/a
Burghardt et al.	2008	case report	Germany	single	2008	1	22 (0)	66 (0)	n/a

**Table 2:** Statistical analysis results of primary and secondary outcomes. Model results and heterogeneity.

	Model	Metric	Estimate	Lower Bound	Upper Bound	Std. Error	*p-Value*	*Tau^2^*	*Q*	*Het. p-Value*	*I^2^*
**Primary outcome**											
**Overall Complications**	BF-E	Proportion	0.219	0.107	0.332	0.05	<0.001	0	0.18	0.99	0
**Secondary outcome**											
**Intraoperative Complications**	BF-E	Proportion	0.199	0.094	0.303	0.053	<0.001	0	3.19	0.67	0
**Intraoperative Hemorrhage**	BF-E	Proportion	0.097	0.02	0.175	0.04	0.014	0	2.691	0.747	0
**Intraoperative Blood Loss (mL)**	CF-E	Mean	29.9	26.42	33.57	1.826	<0.001	0	0.001	0.972	0
**Intraoperative Bladder Injury**	BF-E	Proportion	0.104	0.022	0.185	0.042	0.013	0	0.755	0.98	0
**Conversion**	BF-E	Proportion	0.053	−0.006	0.112	0.03	0.076	0	2.841	0.725	0
**Hematoma**	BF-E	Proportion	0.049	−0.008	0.106	0.029	0.093	0	1.851	0.869	0
**Ileus**	BF-E	Proportion	0.062	−0.032	0.155	0.048	0.196	0	1.406	0.843	0
**Vaginal Infection**	BF-E	Proportion	0.109	−0.016	0.234	0.064	0.088	0	0.744	0.946	0
**Bacteremia**	BF-E	Proportion	0.109	−0.016	0.234	0.064	0.088	0	0.744	0.946	0
**Anastomotic Bleeding**	BF-E	Proportion	0.062	−0.032	0.155	0.048	0.196	0	1.406	0.843	0
**Recurrence**	BF-E	Proportion	0.062	−0.032	0.155	0.048	0.196	0	1.406	0.843	0
**Operation Duration (minutes)**	CF-E	Mean	176.42	170.76	182.08	2.88	<0.001	0	1.348	0.510	0
**Length of Hospital Stay (days)**	CR-E	Mean	8.68	3.29	14.07	2.74	0.002	14.7	37.2	<0.001	97.3
**Lymph Node Yield**	CR-E	Mean	20.6	15.2	25.9	2.74	<0.001	14	13.8	<0.001	92.8

## Abbreviations

The following abbreviations are used in this manuscript:

NOTES	Natural Orifice Translumenal Endoscopic Surgery
TaTME	Trans-Anal Total Mesorectal Excision
NOSE	Natural Orifice Specimen Extraction
vNOTES	Transvaginal NOTES
PRISMA	Preferred Reporting Items for Systematic Reviews and Meta-Analyses
NHLBI	National Heart, Lung, And Blood Institute
SD	Standard Deviation
IQR	Interquartile Range
95% CI	95% Confidence Interval
RP	Raw Proportion
RE	Random Effects
FE	Fixed-Effect
CF-E	Continuous Fixed-Effect
CR-E	Continuous Random-Effect
BF-E	Binary Fixed-Effect
n/a	Not Applicable
BMI	Body Mass Index
ASA	American Society of Anesthesiologists
TNM	Tumor Node Metastasis

## Appendix A

**Table A1:** Patient and Tumor Characteristics.

First Author	Previous Abdominal Operations	ASA			T					N			M	Tumour Location		
		I	II	III	Tis	1	2	3	4	0	1	2	0	Cecum/Ascending	Hepatic Flexure/ Transverse	Tumor Size (cm)
Seow-en et al.	0	0		1	0				1	0	1	0	1	1	0	3
Xiao et al.	n/a	n/a			n/a									n/a	n/a	n/a
Song et al.	0	0	1	0	0		1	0		1	0		0	1	0	2.5 (0)
Xiao et al.	n/a	10	2	0	1	2	1	2	3	8	4	0	5	n/a	n/a	6.75 (1.5)
Bullian et al.	n/a	1	1	2	n/a	n/a	n/a	n/a	n/a	n/a	n/a	n/a	n/a	n/a	n/a	n/a
Burghardt et al.	1	0	1	0	0					1	0		1	1	0	n/a

**Table A2:** Operative Technique Characteristics.

First Author	Bowel Preparation	Position	Pneumoperitoneum (mmHg)	Access	Laparoscopic/Robotic	Trocar		Anastomosis	Approach	Number of Surgeons	Access Closure
						Abdominal	Vaginal				
Seow-en et al.	no	lithotomy & trendelenburg	12	wound protector & glove	lap	1	4	intracorporeal antiperistaltic stapled side-to-side	inferior to superior		direct suture
Xiao et al.	n/a	lithotomy	14	Single Port Access System HTKD Medical™	lap	0	5	intracorporeal stapled antiperistaltic side-to-side	caudal	n/a	n/a
Song et al.	mechanical & oral antibiotics	lithotomy & trendelenburg	n/a	StarPort™	lap	0	3	extracorporeal stapled end-to-side	caudal	n/a	direct suture
Xiao et al.	n/a	lithotomy	14	Single Port Access System HTKD Medical™	lap	0	5	intracorporeal stapled antiperistaltic side-to-side	caudal	n/a	n/a
Bullian et al.	n/a	n/a	n/a	n/a	lap	n/a	n/a	n/a	n/a	n/a	direct suture
Burghardt et al.	n/a	lithotomy	n/a	12mm trocar	lap	2	1	intracorporeal stapled side-to-side	n/a	n/a	n/a

**Table A3:** Quality Assessment Checklist for Case Reports.

	Seow-en et al. (2024)	Song et al. (2021)	Burghardt et al. (2008)
Q1. Were the patient’s demographic characteristics clearly described?	YES	NO	NO
Q2. Was the patient’s history clearly described and presented as a timeline?	YES	YES	YES
Q3. Was the current clinical condition of the patient clearly described?	YES	YES	YES
Q4. Were diagnostic tests or assessment methods and the results clearly described?	YES	YES	YES
Q5. Was the intervention(s) or treatment procedure(s) clearly described?	YES	YES	YES
Q6. Was the post-intervention clinical condition clearly described?	YES	YES	YES
Q7. Were adverse events (harms) or unanticipated events identified and described?	YES	YES	YES
Q8. Does the case report provide takeaway lessons?	YES	YES	YES

**Table A4:** Quality Assessment Checklist for Cohorts.

	Xiao et al. (2023)	Xiao et al. (2020)	Bullian et al. (2014)
1. Was the research question or objective in this paper clearly stated?	YES	YES	YES
2. Was the study population clearly specified and defined?	YES	YES	YES
3. Was the participation rate of eligible people at least 50%?	YES	YES	YES
4. Were all the subjects selected or recruited from the same or similar populations (including the same period)? Were inclusion and exclusion criteria for being in the study prespecified and applied uniformly to all participants?	YES	YES	NO
5. Was sample size justification, power description, or variance and effect estimates provided?	YES	YES	NO
6. For the analyses in this paper, were the exposure(s) of interest measured prior to the outcome(s) being measured?	YES	YES	YES
7. Was the timeframe sufficient so that one could reasonably expect to see an association between exposure and outcome if it existed?	YES	YES	YES
8. For exposures that can vary in amount or level, did the study examine different levels of exposure as related to the outcome (e.g., categories of exposure, or exposure measured as continuous variable)?	YES	YES	YES
9. Were the exposure measures (independent variables) clearly defined, valid, reliable, and implemented consistently across all study participants?	YES	YES	YES
10. Was the exposure(s) assessed more than once over time?	NO	NO	NO
11. Were the outcome measures (dependent variables) clearly defined, valid, reliable, and implemented consistently across all study participants?	YES	YES	YES
12. Were the outcome assessors blinded to the exposure status of participants?	YES	YES	NO
13. Was loss to follow-up after baseline 20% or less?	NO	NO	NO
14. Were key potential confounding variables measured and adjusted statistically for their impact on the relationship between exposure(s) and outcome(s)?	YES	YES	YES

**Table A5:** Quality Assessment Scores.

Study	Study Type	Total Items Assessed	Items Answered “Yes”	Quality Score (%)
Seow-en et al. (2024)	Case Report	8	8	100
Song et al. (2021)	Case Report	8	7	87.5
Burghardt et al. (2008)	Case Report	8	7	87.5
Xiao et al. (2020)	Cohort	14	12	85.7
Xiao et al. (2023)	Cohort	14	12	85.7
Bullian et al. (2014)	Cohort	14	11	78.6

## References

[1] Kim H.S., Noh G.T., Chung S.S., Lee R.A. (2023). Long-Term Oncological Outcomes of Robotic versus Laparoscopic Approaches for Right Colon Cancer: A Systematic Review and Meta-Analysis. Tech. Coloproctol..

[2] Kossenas K., Moutzouri O., Georgopoulos F. (2025). Comparison of Short-Term Outcomes of Robotic versus Laparoscopic Right Colectomy for Patients ≥ 65 Years of Age: A Systematic Review and Meta-Analysis of Prospective Studies. J. Robot. Surg..

[3] Rausa E., Bianco F., Kelly M.E., Aiolfi A., Petrelli F., Bonitta G., Sgroi G. (2019). Systemic Review and Network Meta-Analysis Comparing Minimal Surgical Techniques for Rectal Cancer: Quality of Total Mesorectum Excision, Pathological, Surgical, and Oncological Outcomes. J. Surg. Oncol..

[4] Mostafa O.E.S., Zaman S., Beedham W., Kakaniaris G., Husain N., Kumar L., Akingboye A., Waterland P. (2024). Systematic Review and Meta-Analysis Comparing Outcomes of Multi-Port versus Single-Incision Laparoscopic Surgery (SILS) in Hartmann’s Reversal. Int. J. Colorectal Dis..

[5] Brucchi F., Montroni I., Cirocchi R., Taffurelli G., Vitellaro M., Mascianà G., Sandri G.B.L., Dionigi G., Lauricella S. (2025). A Systematic Review of the Da Vinci^®^ Single-Port System (DVSP) in the Context of Colorectal Surgery. Int. J. Colorectal Dis..

[6] Pompeu B.F., Guerra L.S., de Guedes L.S.S.P., Brunini J.H., Delgado L.M., Poli de Figueiredo S.M., Formiga F.B. (2025). Natural Orifice Extraction Techniques (Natural Orifice Specimen Extraction and Natural Orifice Transluminal Endoscopic Surgery) for Left-Sided Colorectal Cancer: A Systematic Review and Meta-Analysis of Randomized Controlled Trials. J. Laparoendosc. Adv. Surg. Tech. A.

[7] Lee G.C., Sylla P. (2015). Shifting Paradigms in Minimally Invasive Surgery: Applications of Transanal Natural Orifice Transluminal Endoscopic Surgery in Colorectal Surgery. Clin. Colon. Rectal Surg..

[8] Fuchs K.H., Meining A., Von Renteln D., Fernandez-Esparrach G., Breithaupt W., Zornig C., Lacy A. (2013). Euro-NOTES Status Paper: From the Concept to Clinical Practice. Surg. Endosc..

[9] Bulian D.R., Runkel N., Burghardt J., Lamade W., Butters M., Utech M., Thon K.P., Lefering R., Heiss M.M., Buhr H.J. (2014). Natural Orifice Transluminal Endoscopic Surgery (NOTES) for Colon Resections—Analysis of the First 139 Patients of the German NOTES Registry (GNR). Int. J. Colorectal Dis..

[10] Marks J.H., Montenegro G.A., Salem J.F., Shields M.V., Marks G.J. (2016). Transanal TATA/TME: A Case-Matched Study of TaTME versus Laparoscopic TME Surgery for Rectal Cancer. Tech. Coloproctol..

[11] Stipa F., Burza A., Curinga R., Santini E., Delle Site P., Avantifiori R., Picchio M. (2015). Laparoscopic Colon and Rectal Resections with Intracorporeal Anastomosis and Trans-Vaginal Specimen Extraction for Colorectal Cancer. A Case Series and Systematic Literature Review. Int. J. Colorectal Dis..

[12] Efetov S.K., Cao Y., Panova P.D., Khlusov D.I., Shulutko A.M. (2024). Reduced-Port Laparoscopic Right Colonic Resection with D3 Lymph Node Dissection and Transvaginal Specimen Extraction (NOSES VIIIa) for Right Colon Cancer: Clinical Features. Tech. Coloproctol..

[13] Moloney J.M., Gan P.S.L. (2016). Hybrid Transvaginal NOTES and Mini-Laparoscopic Colectomy: Benefit Through Synergy. JSLS.

[14] Park J.S., Choi G.S., Lim K.H., Jang Y.S., Kim H.J., Park S.Y., Jun S.H. (2010). Clinical Outcome of Laparoscopic Right Hemicolectomy with Transvaginal Resection, Anastomosis, and Retrieval of Specimen. Dis. Colon. Rectum.

[15] Xiao Y., Lin C., Lu J.Y., Xu L., Hou W.Y., Sun R., Chang G.J., Zhang J.J. (2021). Short-Term Outcomes of Pure Transvaginal Laparoscopic Right Colectomy: A Novel Surgery Approach Based on an Idea, Development, Exploration, Assessment, Long-Term Framework Stage IIa Study. J. Surg. Oncol..

[16] Higgins J.P.T., Thomas J., Chandler J., Cumpston M., Li T., Page M.J., Welch V.A. (2024). Cochrane Handbook for Systematic Reviews of Interventions Version 6.5 (Updated August 2024).

[17] Page M.J., McKenzie J.E., Bossuyt P.M., Boutron I., Hoffmann T.C., Mulrow C.D., Shamseer L., Tetzlaff J.M., Akl E.A., Brennan S.E. (2021). The PRISMA 2020 Statement: An Updated Guideline for Reporting Systematic Reviews. BMJ.

[18] Hozo S.P., Djulbegovic B., Hozo I. (2005). Estimating the Mean and Variance from the Median, Range, and the Size of a Sample. BMC Med. Res. Methodol..

[19] Xiao Y., Sun Z., Sun R., Hou W., Xu L., Lu J. (2023). Safety and Feasibility of Right Colectomy via a Transvaginal Approach: Early Experience from a Single Center. Zhonghua Wei Chang Wai Ke Za Zhi.

[20] Burghardt J., Federlein M., Müller V., Benhidjeb T., Elling D., Gellert K. (2008). Minimal Invasive Transvaginal Right Hemicolectomy: Report of the First Complex NOS (Natural Orifice Surgery) Bowels Operation Using a Hybrid Approach. Zentralbl Chir..

[21] Song Z.J., Shi Y.Q., Jiang Y.M., Liu K., Li Y., Wang C.G., Zhao R. (2021). Pure Transvaginal Natural Orifice Transluminal Endoscopic Surgery Right Hemicolectomy for Colon Cancer: A Case Report. World J. Clin. Cases.

[22] Seow-En I., Villanueva M.E., Seah A.W.M., Tan E.J.K.W., Ang J.X. (2024). Vaginal Natural Orifice Transluminal Endoscopic Surgery (VNOTES) Right Hemicolectomy with Intracorporeal Anastomosis for Cecal Cancer. Tech. Coloproctol..

[23] Gu C., Wu Q., Zhang X., Wei M., Wang Z. (2021). Single-Incision versus Conventional Multiport Laparoscopic Surgery for Colorectal Cancer: A Meta-Analysis of Randomized Controlled Trials and Propensity-Score Matched Studies. Int. J. Colorectal Dis..

[24] Li C.B., Hua K.Q. (2020). Transvaginal Natural Orifice Transluminal Endoscopic Surgery (VNOTES) in Gynecologic Surgeries: A Systematic Review. Asian J. Surg..

[25] Shen X., Zhou C., Hua Q., Yang L., Zhao W., Xu P. (2022). Impact of Operation Duration on Short-Term and Long-Term Prognosis in Patients Undergoing Radical Colorectal Surgery. J. Cancer.

[26] Rausa E., Kelly M.E., Asti E., Aiolfi A., Bonitta G., Bonavina L. (2019). Right Hemicolectomy: A Network Meta-Analysis Comparing Open, Laparoscopic-Assisted, Total Laparoscopic, and Robotic Approach. Surg. Endosc..

[27] Liu X., Yang W.H., Jiao Z.G., Zhang J.F., Zhang R. (2019). Systematic Review of Comparing Single-Incision versus Conventional Laparoscopic Right Hemicolectomy for Right Colon Cancer. World J. Surg. Oncol..

[28] Migliore M., Giuffrida M.C., Marano A., Pellegrino L., Giraudo G., Barili F., Borghi F. (2021). Robotic versus Laparoscopic Right Colectomy within a Systematic ERAS Protocol: A Propensity-Weighted Analysis. Updates Surg..

[29] Solaini L., Bazzocchi F., Cavaliere D., Avanzolini A., Cucchetti A., Ercolani G. (2018). Robotic versus Laparoscopic Right Colectomy: An Updated Systematic Review and Meta-Analysis. Surg. Endosc..

[30] Apostolou K.G., Orfanos S.V., Papalois A.E., Felekouras E.S., Zografos G.C., Liakakos T. (2015). Single-Incision Laparoscopic Right Hemi-Colectomy: A Systematic Review. Indian J. Surg..

[31] Chang J.H.E., Xu H., Zhao Y., Wee I.J.Y., Ang J.X., Tan E.K.W., Seow-En I. (2024). Transvaginal versus Transabdominal Specimen Extraction in Minimally Invasive Surgery: A Systematic Review and Meta-Analysis. Langenbecks Arch. Surg..

[32] Tsai P.L., Chen J.S., Lin C.H., Hsu T.C., Lin Y.W., Chen M.J. (2024). Abdominal Wound Length Influences the Postoperative Serum Level of Interleukin-6 and Recovery of Flatus Passage among Patients with Colorectal Cancer. Front. Surg..

[33] Bartels S.A.L., Vlug M.S., Ubbink D.T., Bemelman W.A. (2010). Quality of Life after Laparoscopic and Open Colorectal Surgery: A Systematic Review. World J. Gastroenterol..

[34] Nakamura T., Takayama Y., Sato T., Watanabe M. (2020). Risk Factors for Wound Infection After Laparoscopic Surgery for Colon Cancer. Surg. Laparosc. Endosc. Percutan Tech..

[35] Leung A.L.H., Cheung H.Y.S., Fok B.K.L., Chung C.C.C., Li M.K.W., Tang C.N. (2013). Prospective Randomized Trial of Hybrid NOTES Colectomy Versus Conventional Laparoscopic Colectomy for Left-Sided Colonic Tumors. World J. Surg..

[36] He J., Hu J.F., Shao S.X., Yao H.B., Zhang X.F., Yang G.G., Shen Z. (2020). The Comparison of Laparoscopic Colorectal Resection with Natural Orifice Specimen Extraction versus Mini-Laparotomy Specimen Extraction for Colorectal Tumours: A Systematic Review and Meta-Analysis of Short-Term Outcomes. J. Oncol..

[37] Kapurubandara S., Lowenstein L., Salvay H., Herijgers A., King J., Baekelandt J. (2021). Consensus on Safe Implementation of Vaginal Natural Orifice Transluminal Endoscopic Surgery (VNOTES). Eur. J. Obstet. Gynecol. Reprod. Biol..

[38] Clark J., Sodergren M., Noonan D., Darzi A., Yang G.Z. (2009). The Natural Orifice Simulated Surgical Environment (NOSsE): Exploring the Challenges of NOTES without the Animal Model. J. Laparoendosc. Adv. Surg. Tech. A.

[39] Gillen S., Gröne J., Knödgen F., Wolf P., Meyer M., Friess H., Buhr H.-J., Ritz J.-P., Feussner H., Lehmann K.S. (2012). Educational and Training Aspects of New Surgical Techniques: Experience with the Endoscopic–Laparoscopic Interdisciplinary Training Entity (ELITE) Model in Training for a Natural Orifice Translumenal Endoscopic Surgery (NOTES) Approach to Appendectomy. Surg. Endosc..

[40] Wang C.J., Go J., Huang H.Y., Wu K.Y., Huang Y.T., Liu Y.C., Weng C.H. (2019). Learning Curve Analysis of Transvaginal Natural Orifice Transluminal Endoscopic Hysterectomy. BMC Surg..

[41] Korzeniowski P., Barrow A., Sodergren M.H., Hald N., Bello F. (2016). NOViSE: A Virtual Natural Orifice Transluminal Endoscopic Surgery Simulator. Int. J. Comput. Assist. Radiol. Surg..

[42] Lau S.Y.C., Choy K.T., Yang T.W.W., Heriot A., Warrier S.K., Guest G.D., Kong J.C. (2022). Defining the Learning Curve of Transanal Total Mesorectal Excision: A Systematic Review and Meta-Analysis. ANZ J. Surg..

